# hiPSCGEM01: A Genome-Scale Metabolic Model for Fibroblast-Derived Human iPSCs

**DOI:** 10.3390/bioengineering12101128

**Published:** 2025-10-21

**Authors:** Anna Procopio, Elvira Immacolata Parrotta, Stefania Scalise, Paolo Zaffino, Rita Granata, Francesco Amato, Giovanni Cuda, Carlo Cosentino

**Affiliations:** 1 Department of Experimental and Clinical Medicine, Università degli Studi Magna Græcia, 88100 Catanzaro, Italy; 2 Department of Medical and Surgical Sciences, Università degli Studi Magna Græcia, 88100 Catanzaro, Italy; 3 Department of Electrical Engineering and Information Technology, Università di Napoli, Federico II, 80125 Napoli, Italy

**Keywords:** induced pluripotent stem cells, genome-scale metabolic model, flux-balance analysis, essential metabolites

## Abstract

Human induced pluripotent cells (hiPSCs), generated in vitro, represent a groundbreaking tool for tissue regeneration and repair. Understanding the metabolic intricacies governing hiPSCs is crucial for optimizing their performance across diverse environmental conditions and improving production strategies. To this end, in this work, we introduce *hiPSCGEM01*, the first genome-scale, context-specific metabolic model (GEM) uniquely tailored to fibroblast-derived hiPSCs, marking a clear distinction from existing models of embryonic and cancer stem cells. *hiPSCGEM01* was developed using relevant genome expression data carefully selected from the Gene Expression Omnibus (GEO), and integrated with the RECON 3D framework, a comprehensive genome-scale metabolic model of human metabolism. Redundant and unused reactions and genes were identified and removed from the model. Key reactions, including those facilitating the exchange and transport of metabolites between extracellular and intracellular environments, along with all metabolites required to simulate the growth medium, were integrated into *hiPSCGEM01*. Finally, blocked reactions and dead-end metabolites were identified and adequately solved. Knockout simulations combined with flux balance analysis (FBA) were employed to identify essential genes and metabolites within the metabolic network, providing a comprehensive systems-level view of fibroblast-derived hiPSC metabolism. Notably, the model uncovered the unexpected involvement of nitrate and xenobiotic metabolism—pathways not previously associated with hiPSCs—highlighting potential novel mechanisms of cellular adaptation that merit further investigation. *hiPSCGEM01* establishes a robust platform for in silico analysis and the rational optimization of in vitro experiments. Future applications include the evaluation and refinement of culture media, the design of new formulations, and the prediction of hiPSC responses under varying growth conditions, ultimately advancing both experimental and clinical outcomes.

## 1. Introduction

Cellular therapies hold significant promise in addressing debilitating and degenerative disorders marked by progressive tissue deterioration. This cutting-edge approach paves the way for novel therapeutic strategies, offering innovative solutions to some of the most pressing medical challenges [1,2,3,4,5]. Advanced therapies frequently involve the transplantation of selected and laboratory-processed stem cells into patients to repair and restore organ or tissue function. This highlights the transformative potential of stem cells in modern medicine. In particular, pluripotent stem cells (PSCs) have shown significant promise in clinical applications, with numerous studies emphasizing their role, especially in liver regeneration. Given the liver’s vulnerability to a wide range of diseases, many of which are severe or life-threatening [6], PSCs represent a crucial avenue for developing innovative treatments. This can be achieved using liver-specific stem cells, such as hepatic progenitor cells (HPCs) [7,8], or mesenchymal stem cells (MSCs), derived from bone marrow, adipose tissue, or umbilical cord blood [9,10]. Stem cells can differentiate into various live-specific cell types, including hepatocytes, critical for metabolism, protein synthesis, and detoxification, and cholangiocytes and stellate cells, which play key roles in bile duct development and fibrosis regulation. Among the different stem cell types, PSCs stand out due to their extraordinary ability to differentiate into a wide range of cell types, making them particularly promising for cellular therapies and regenerative medicine. While embryonic stem cells (ESCs), derived from the early stages of developing embryos, have the potential to differentiate into any cell type of the body, their use is limited by ethical concerns, and the risk of post-implantation rejection [11]. This has shifted the focus toward alternative sources like induced pluripotent stem cells (iPSCs), which offer pluripotency without the ethical concerns linked to ESCs. iPSCs are generated by reprogramming adult cells such as fibroblasts into a pluripotent state, by introducing specific transcription factors, such as Oct3/4, c-Myc, Klf4, and Sox2, collectively known as Yamanaka factors [12]. This breakthrough has revolutionized the field, offering a versatile and ethically acceptable option for regenerative medicine. This process eliminates the risk of immune rejection since iPSCs share the same genetic background as the patient. As a result, the study, laboratory creation, and clinical application of human iPSCs (hiPSCs) pave the way for personalized medicine. A deep understanding of hiPSCs metabolism is fundamental to optimizing their therapeutic potential and ensuring their effectiveness in clinical applications. Metabolism is crucial for maintaining the key characteristics of hiPSCs, including pluripotency, differentiation, and essential cellular functions. Investigating and manipulating metabolic pathways can enhance reprogramming efficiency, influence differentiation outcomes, and optimize the overall functionality of hiPSCs for therapeutic applications.

**Figure 1:**
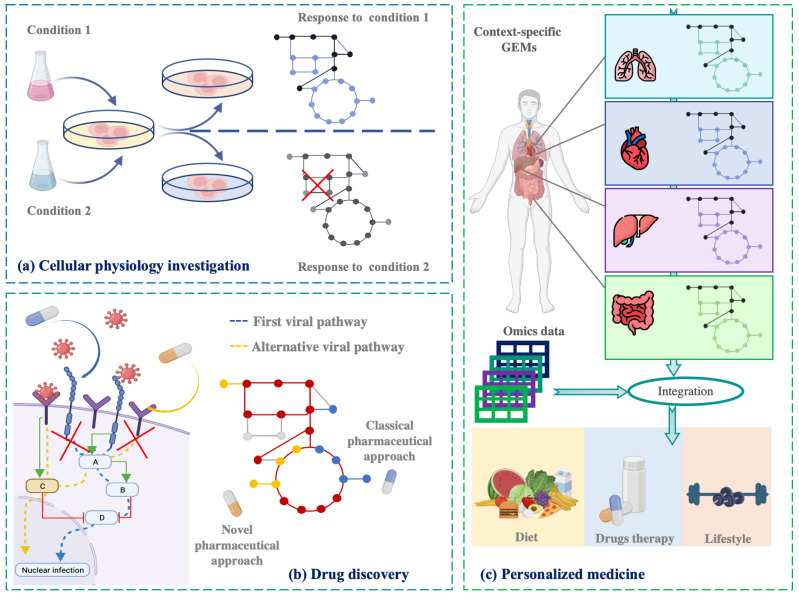
Graphical representation of the main applications fields of the GEMs that can help (**a**) to understand cellular physiology and how the cells respond to different experimental conditions or different growth conditions; (**b**) to identify the new therapeutic targets and design new optimized drugs; (**c**) to personalize the therapeutic plan, the diet, and the lifestyle of a single patient. Figure created using the BioRender online platform (https://app.biorender.com/).

Systems biology offers an efficient, cost-effective, and accurate approach to conducting a variety of experiments and in-depth studies on hiPSCs. Specifically, genome-scale metabolic models (GEMs) provide a comprehensive framework to explore the diverse cellular metabolic aspects [13], going beyond the limitations of traditional mathematical models [14,15,16,17,18,19,20,21]. Moreover, GEMs encompass several medical and biological fields, ranging from the simple investigation of unknown cellular aspects to the identification of novel therapeutic targets for drug discovery and personalized medicine [22,23], as reported in Figure 1. Analyzing the metabolic changes in both healthy and diseased provides valuable insights into the underlying mechanisms of disease progression. Several GEM applications are focused on studying the human microbiota, aiming to understand the key metabolic products and the interactions between microbial colonies that contribute to various physiological and pathological conditions [24,25,26]. Furthermore, a well-structured GEM offers a valuable tool for investigating the metabolic dynamics underlying cancer development, contributing to our understanding of the tumor microenvironment, the processes beyond the exchange of metabolites between cancer cells and stromal cells, the influence of nutrient availability and oxygen levels on tumor growth and its proliferation [27,28], and the involved pathways and the essential genes [29,30]. GEMs also offer significant contributions to drug development by simulating and analyzing the effects of specific pharmacological therapies. This in silico approach aids in the optimization of treatment strategies through the identification of more specific and effective drug targets [31,32]. Emerging research areas now explore the synergy between genome-scale metabolic models and artificial intelligence techniques to overcome the limitations of individual methods, leading to significant advancements in outcomes [33]. Notably, Magazzù et al. [34] proposed a multi-omics pipeline combining computational modeling and machine learning techniques to explore the underlying mechanisms of hepatoblastoma to improve diagnostic processes. Expanding the application of GEMs, these models are also being used to investigate the metabolic differences in neuropsychiatric disorders such as schizophrenia, bipolar disorder, and depression [35]. In the field of stem cells, Barata et al. [36] have proposed a genome-scale metabolic model to scrutinize the metabolic aspects of cancer stem cells (CSC) and identify potential therapeutic targets.

From a mathematical point of view, a GEM is a computational framework that represents the complete set of metabolic pathways and reactions in an organism based on its annotated genome. GEMs are widely used in systems biology to simulate, analyze, and predict the metabolic capabilities of cells, tissues, or entire organisms [37,38]. Given their significant utility, efforts have been made to develop robust algorithms for the reconstruction and validation of these models. These efforts leverage omics data, including transcriptomic and proteomic data, as well as existing knowledge from the scientific literature, to build and refine accurate GEMs [39].

In this work, we provide the first version of a dedicated GEM specific to fibroblast-derived hiPSCs. The existing GEMs primarily focus on other cell types, such as cancer stem cells, embryonic stem cells (ESCs), or non-human models. hiPSCGEM01 represents a novel contribution as it is specifically tailored to hiPSCs derived from fibroblast cells. Additionally, our model addresses specific metabolic pathways that are underexplored in this context, such as those involved in nitrate and xenobiotic metabolism, which could be important for metabolic adaptation and the functionality of the cells under various growth conditions. Therefore, compared to existing models, our model offers a more focused and context-specific approach for hiPSCs derived from fibroblasts, filling an important gap in the field and providing a valuable tool for future studies and optimizing culture conditions and cell functionality for clinical applications. The reconstruction, assessment, and validation of this model, as outlined in Section 2 of the work, are crucial steps in ensuring its accuracy and reliability. The analysis of the main reconstructed pathways and essential genes are described in Section 3, while a discussion of these findings and their implications is provided in Section 4.

## 2. Materials & Methods

### 2.1. Constraint-Based Models and Flux-Balance Analysis

**Figure 2:**
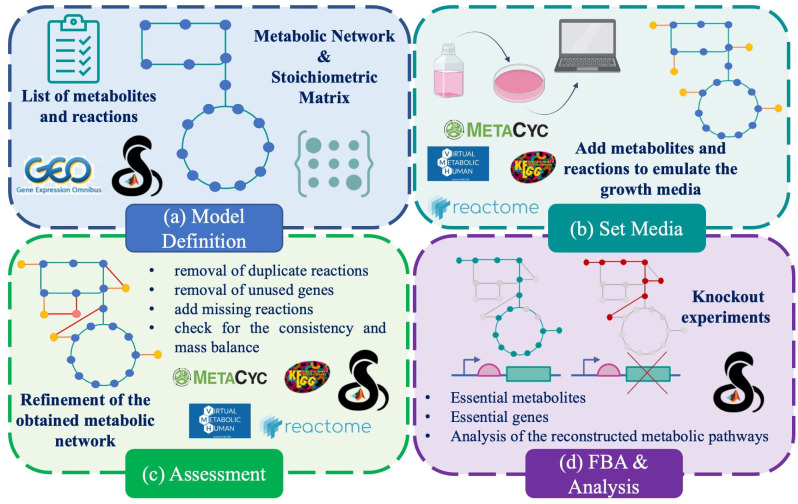
Graphical representation of the main steps followed to reconstruct and validate the hiPSCGEM01: (**a**) Selection of appropriate transcription data and reconstruction of a raw GEM from RECON3D; (**b**) setting of appropriate conditions to emulate the growth medium typical for the hiPSC cells; (**c**) curation and assessment of the devised model; (**d**) design of appropriate FBA-based experiments to investigate the assessed model.

The reconstruction of the proposed GEM for fibroblast-derived human iPSCs was performed by integrating curated biochemical databases, computational algorithms, and manual refinement to obtain a context-specific metabolic network (Figure 2). Based on stoichiometric matrices, GEMs enable constraint-based modeling [37], where the fluxes of metabolic reactions are constrained according to experimental or physiological data. Quality control included verification of annotation–reaction consistency, thermodynamic feasibility, and transport and exchange reactions [40]. This framework allowed the systematic analysis of metabolic fluxes, identification of network bottlenecks, and prediction of optimal metabolic states [41]. Subsequently, flux balance analysis (FBA) [42] was applied to quantitatively evaluate the reconstructed metabolic network. The stoichiometric matrix *S* defines the mass-balance system:

(1) $$\frac{dx}{dt}=S\times \nu$$

where ν represents the flux vector and $x={\left(x_{1},x_{2},\ldots ,x_{m}\right)}^{T}$ denotes metabolite concentrations. Under steady-state conditions, the system reduces to the following:

(2a) $$\begin{array}{cc} S\times \nu & =0 \end{array}$$

(2b) $$\begin{array}{cc} a_{i}\leq \nu_{i} & \leq b_{i} \end{array}$$

Generally, this system is underdetermined, and biologically feasible flux distributions are obtained by imposing physicochemical and experimental constraints. Optimization algorithms are then employed to maximize a selected objective function, such as biomass or ATP yield, to identify flux distributions that best represent the biological behavior of the modeled system [43].

In this work, the biomass production reaction was defined as the primary objective function, as it effectively represents the growth and proliferation potential of hiPSCs under varying culture and environmental conditions [44]. By optimizing this function, the model simulates the metabolic activity of hiPSCs and enables comparative analyses under different nutrient and culture scenarios, providing a robust framework for in silico investigation of stem cell metabolism.

### 2.2. Data Pre-Processing

The context-specific network for hiPSCs was obtained by exploiting the genome expression data of human fibroblasts properly selected from the Gene Expression Omnibus (GEO) databank. As detailed in Table 1, the dataset comprised ten hiPSC samples generated from human fibroblasts using the Affymetrix HG-U133 2 Plus platform.Gene expression levels were evaluated based on hybridization intensity, categorized as Present (P), Absent (A), or Marginal (M) for each probe set [45] and previously calculated by the authors of the reference papers [46,47].

Subsequently, the ubiquity score, indicating the frequency with which a gene is expressed in a collection of relevant samples, was calculated for each gene as described in [48]. This metric was determined by binarizing gene expression levels (1 for the Present call, and 0 otherwise) and applying the following function:

(3) $$U_{g}=\frac{\sum_{n\in N}X_{g_{n}}}{N}$$

where *X* represents the binary value associated with the gene *g* in the experiment *n*, while *N* is the total number of samples. The pre-processing phase, including the labels evaluation, and the computation of the ubiquity score, was performed in R (version 4.3.2) by using the Bioconductor (version 3.21) [49], and in the MATLAB (version R2024b) environment.

### 2.3. Model Reconstruction

We obtained the specific GEM for the hiPSCs cells by exploiting both the *mCADRE* (metabolic Context-specificity Assessed by Deterministic Reaction Evaluation) [48], and the COBRA toolbox functions [50]. The decision to employ mCADRE for the reconstruction of *hiPSCGEM01* was primarily driven by its capacity to efficiently integrate diverse transcriptomic datasets and its ability to generate highly detailed metabolic models. As input, mCADRE required gene expression data and the comprehensive human metabolic model, Recon3D [51].

To prepare the resulting GEMs, *hiPSCGEM01* for flux balance analysis (FBA), we incorporated essential metabolites and exchange reactions required to simulate the hiPSC growth medium, as detailed below.

### 2.4. Adaptation to Culture Medium

One of the most attractive characteristics of the metabolic models concerns the possibility to simulate different experimental conditions simply by defining appropriate constraints. In fact, by modifying the lower and upper bounds associated with each metabolic modeled reaction, it is possible to observe how cellular metabolic phenotypes respond to variations in media composition, including those mimicking pathological states. This approach enables the simulation of cellular metabolic responses to specific metabolite availability both in terms of growth rate and product formation.

In this regard, first of all, we simulated standard hiPSC culture conditions using mTeSR medium, [52]. To establish the medium conditions in our computational model, metabolite concentrations were converted to corresponding metabolic fluxes by dividing by molecular weight. These calculated fluxes were subsequently applied as lower bounds for relevant exchange reactions. Furthermore, to build a comprehensive and robust metabolic model, we systematically incorporated all needed metabolites and missing exchange and transport reactions by drawing upon information gathered from authoritative online databases, including but not limited to MetaCyc [53], Kyoto Encyclopedia of Genes and Genomes (KEGG) [54], Virtual Human Metabolism (VHM) [55], Reactome [56], and the atlas of human metabolism, HUMAN1 [57].

All computational tasks, including flux calculations, lower and upper bound definition, and incorporation of needed metabolites and transport/exchange reactions, were executed using the COBRA Toolbox [58].

### 2.5. Consistency Testing and Model Validation

Before FBA, the reconstructed network underwent rigorous validation to ensure structural consistency, as outlined in [40].

The validation process involved a thorough examination of the network to identify and remove unused genes and redundant reactions. Additionally, metabolite information was comprehensively evaluated, including parameters such as molecular charge, chemical formula, and cross-referencing with databases like KEGG and HMDB [59]. In particular, the curation of all metabolite data ensured stoichiometric consistency. A stoichiometrically consistent network adheres to mass conservation principles, guaranteeing a balance between metabolite production and consumption within the system [60].

Then, to ensure the robustness of the hiPSCGEM01 model for subsequent analyses, a validation process was undertaken to identify and eventually eliminate dead-end metabolites (DEMs) and blocked reactions (BRs). This effort was critical to establishing the network’s structural and flux consistency, as these issues could hinder accurate metabolic simulations. The process began with an initial network check conducted by using verifyModel() from the COBRA Toolbox.

DEMs are defined as metabolites that participate in only one reaction within the metabolic network, being either exclusively produced without subsequent consumption or exclusively consumed without prior production by any other known reactions. These metabolites represent isolated components within the metabolic network [61]. The reactions associated with the DEMs are the BRs, and comprise all inactive reactions unable of carrying flux under the given experimental conditions [62]. The presence of DEMs and, consequently, of BRs, determines the inconsistency of the metabolic network from a flux perspective. To identify DEMs and BRs in the metabolic network, the detectDeadEnds() and findBlockedReaction() functions from the COBRA Toolbox were employed. Additionally, flux variability analysis (FVA) [63], a variant of FBA that evaluates the range of allowable fluxes for each reaction under given constraints, was performed to further corroborate the presence of potential BRs.

A final round of validation was performed using MEMOTE [64], a comprehensive evaluation tool for metabolic models. MEMOTE scored the hiPSCGEM01 network for completeness, consistency, and quality. All aforementioned tools confirmed the absence of DEMs and BRs, thereby validating the flux and stoichiometric consistency of the *hiPSCGEM01* model. This established the model’s suitability for subsequent FBA-based analyses.

## 3. Results

The initial *hiPSCGEM01* model, generated by mCADRE, comprised 4038 metabolites, 6024 reactions, and 3697 associated genes. Through an iterative process of model refinement and validation, the model was adapted to specific culture conditions and optimized for accuracy. This process involved the incorporation of additional metabolites and reactions, followed by the removal of unused components. The final refined model consisted of 4295 metabolites, 6012 reactions, and 2773 genes. Essential genes and metabolites were identified by conducting flux balance analysis FBA on this final model.

### 3.1. Analysis and Computation of the Essential Genes

**Figure 3:**
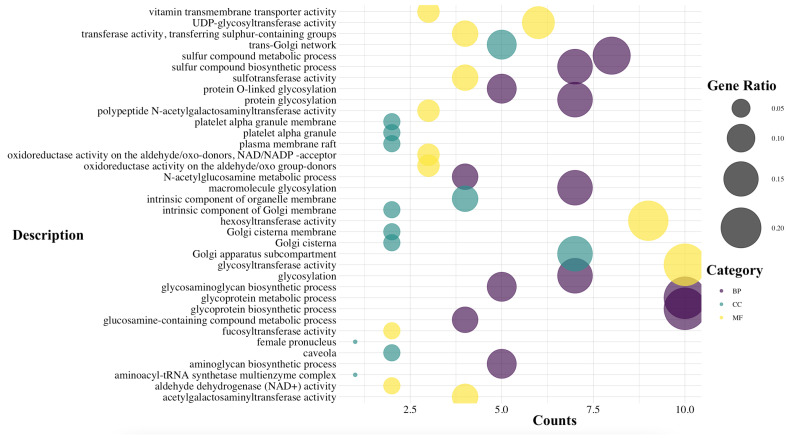
Gene Ontology (GO) analysis conducted on the 14 most significant essential genes, where Gene Ratio represents the percentage of total differentially expressed genes (DEGs) in the given GO term and Counts is the number of the annotated genes in the subset of interest and Molecular Function (MF), Cellular Component (CC), and Biological Process (BP).

Gene essentiality is a pivotal aspect in genetics and molecular biology, as it is intricately intertwined with the fundamental cellular functions and processes imperative for survival, growth, and cellular reproduction [65]. By identifying essential genes, researchers can pinpoint vital metabolic and regulatory pathways, offering insights into both basic biology and potential therapeutic targets. In this study, FBA was used to systematically assess gene essentiality in the hiPSC metabolic network. The processes included simulations involving gene knockouts, performed one gene at a time by removing its corresponding reactions from the model, and subsequently assessed their impact on the cellular growth rate. Using this systematic approach, we identified 764 distinct gene candidates to be essential. Among these, we categorized essential 50 genes whose deletion significantly reduces cellular growth rate. Figure 3 presents the results of the gene ontology enrichment analysis performed on this set of 50 essential genes, categorized into biological process (BP), cellular component (CC), and molecular function (MF).

However, since essential genes are often closely linked to critical cellular pathways, their knockout frequently activates alternative pathways. As a result, accurately assessing the metabolic repercussions of these gene knockouts at the cellular level can be challenging. To address this complexity, especially for future experimental validations, it is beneficial to conduct simulations and detailed investigations of the essential metabolites within the hiPSCs network. All the analyses were performed using the ClusterProfiler [66] package in R.

### 3.2. Analysis and Computation of the Essential Metabolites

Metabolites, as essential intermediates of biological processes, sustain cellular life. Categorized as essential or unessential based on their metabolic role [67], the essential metabolites are those metabolites indispensable for energy production, macromolecular biosynthesis, signal transduction, and cellular homeostasis [68]. Their identification is crucial for understanding cellular metabolism, drug target discovery, and personalized medicine.

To identify essential metabolites critical for hiPSC maintenance, metabolite essentiality analysis was performed. To identify essential metabolites, we individually simulated the complete removal of each metabolite by setting the flux of irreversible reactions consuming it, and the forward flux of reversible reactions utilizing it, to zero. Metabolites were deemed essential if their removal resulted in a ≥50% reduction in biomass production compared to wild-type conditions [69]. Of the 4295 metabolites in *hiPSCGEM01*, 430 were classified as essential.

**Figure 4:**
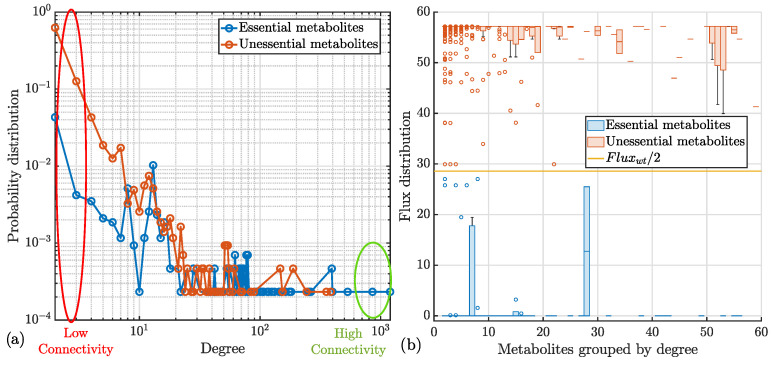
Structural analysis of the essential and unessential metabolites of the network: (**a**) probability distribution computed in the function of the degree of the network; (**b**) distribution of the fluxes obtained from FBA simulating the knockout of each metabolite and grouped by degree. $flux_{wt}$ represents the flux value in the presence of all the 4295 metabolites present in *hiPSCGEM01*. N.B.: the subfigure (**b**) reports a zoom of the first 60-degree groups, since for the other ones the trends of the flux distribution are (i) 0, for the essential metabolites, and (ii) $flux_{wt}$ for the unessential ones.

To explore metabolite connectivity, node degree (number of associated reactions) was calculated for each metabolite. As reported in Figure 4a, the degree distribution for essential and unessential metabolites approximated a power-law distribution [70,71], indicating higher connectivity of essential metabolites. Flux analysis following metabolite knockout revealed that essential metabolites exhibited near-zero flux values, emphasizing their critical role in cellular growth. In contrast, unessential metabolites maintained flux levels similar to wild-type conditions, $flux_{wt}$; see Figure 4b.

To elucidate the metabolic pathways associated with essential metabolites, Metabolite Set Enrichment Analysis (MSEA) was performed using the MetaboAnalyst platform (https://www.metaboanalyst.ca) [72]. The results of MSEA are shown in Table 2.

## 4. Discussion

The developed computational model provides a robust in silico platform for exploring hiPSC metabolism and guiding the formulation of innovative culture media. By applying GO and MSEA to the identified both essential genes and metabolites, we extracted the significantly enriched metabolic pathways, summarized in Figure 3 and in Table 2, respectively. The biological implications in the hiPSCs metabolism of these pathways are discussed below.

### 4.1. Central Metabolic Pathways

A notable focus, highlighted by the FBA conducted on *hiPSCGEM01*, is on lipid metabolism, which plays a fundamental role in hiPSCs biology. Lipids are indispensable not only as structural components of cellular membranes but also as vital energy reservoirs that support self-renewal and maintain pluripotency [73,74,75]. Their critical role in energy production, particularly via fatty acid oxidation, supports hiPSC growth and maturation [76]. Furthermore, de novo fatty acid synthesis, regulated by ACC1 (Acetyl-CoA Carboxylase 1), has been identified as critical for cellular reprogramming [77]. Inhibiting this pathway adversely affects the survival of iPSC-derived cardiomyocytes (iPSC-CM) [78]. Beyond energy production and structural roles, fatty acids also contribute to protein acetylation processes, which significantly influence pluripotency regulation [79,80]. This emphasis on lipid metabolism highlights its intricate connection to cellular functions and underscores its importance as a target for further investigation, both in experimental validation and in the optimization of hiPSCs culture conditions.

Additional metabolic pathways essential for hiPSCs, identified through our in silico analysis, include amino acid, pyruvate, purine, and pyrimidine metabolism. For instance, amino acids such as cysteine and methionine, beyond their role as protein building blocks, are essential for hiPSC growth. Glutathione, synthesized from cysteine, serves as a potent antioxidant, contributing to the maintenance of pluripotency [81]. Moreover, the cystine/glutamate antiporter system is crucial for cysteine uptake and redox balance, ensuring self-renewal and genomic integrity in hiPSCs [82]. Pyruvate metabolism also plays a central role in hiPSCs proliferation. Derived from glycolysis, pyruvate can be metabolized to lactate in anaerobic conditions or converted into acetyl-CoA under aerobic settings [83]. Lactate serves as an alternative carbon source, supporting hiPSC proliferation by facilitating fatty acid synthesis [84,85]. Additionally, purine and pyrimidine metabolism are essential for nucleotide biosynthesis, providing the building block for DNA and RNA. These pathways are integral to energy production, signal transduction, and overall hiPSC proliferation [86,87].

These metabolic pathways collectively contribute to the unique characteristics and functions of hiPSCs. The identification of metabolic pathways closely associated with hiPSC metabolism through *hiPSCGEM01* corroborates its validity and demonstrates its capacity to identify novel pathways that may impact pluripotency and self-renewal.

### 4.2. Emerging Pathways

Beyond these well-established pathways, *hiPSCGEM01* identified additional, less-explored routes potentially relevant to hiPSC biology, including nitrate and xenobiotic metabolism. These pathways may play unrecognized roles in redox regulation, energy balance, and cellular adaptation.

Nitrate metabolism, for example, may indirectly modulate hiPSCs behavior through redox signaling and its interactions with energy metabolism [82,88]. For instance, the metabolic shift from a fibroblast’s oxidative metabolism to the glycolytic metabolism typical of hiPSCs may require nitrate metabolism for regulating nitric oxide (NO) levels, which influence mitochondrial function, energy balance, and cellular signaling during reprogramming [89].

Similarly, xenobiotic metabolism, including pathways like phytanic acid oxidation, ethanol degradation, and nicotinate/nicotinamide metabolism, is essential for cellular homeostasis. The reprogramming of fibroblasts into hiPSCs is a stressful process that generates metabolic byproducts and oxidative stress [90]. Xenobiotic metabolism may play a role in detoxifying harmful compounds produced during this process, ensuring cell survival and the successful establishment of pluripotency. By detoxifying harmful substances, these pathways prevent oxidative stress and cellular damage, both of which can compromise hiPSCs viability and function [91].

Dysregulation of the nitrate and xenobiotic pathways could lead to adverse effects, underscoring the importance of further investigation.

### 4.3. Implication for Regenerative Medicine

Together, these findings demonstrate that hiPSCGEM01 accurately captures core metabolic features of hiPSCs while revealing novel, potentially adaptive pathways that expand our understanding of pluripotent metabolism. The model thus serves as both a validation of known mechanisms and a discovery platform for identifying new targets to optimize culture conditions, enhance self-renewal, and improve regenerative and therapeutic applications.

## 5. Conclusions

To address the knowledge gap in understanding the metabolism of human fibroblast-derived hiPSCs, we developed a context-specific genome-scale metabolic model (GEM), termed *hiPSCGEM01*, tailored to the unique metabolic features of hiPSCs. Through rigorous in silico analyses, including gene knockout and metabolite essentiality studies, the model proved effective in capturing key pathways and identifying critical genes and metabolites essential for hiPSC maintenance. Our findings highlight the central role of lipid and amino acid metabolism, as well as nucleotide biosynthesis, in sustaining hiPSC growth, pluripotency, and homeostasis. Moreover, the identification of underexplored pathways, such as nitrate and xenobiotic metabolism, suggests new directions for research into their indirect influence on stem cell physiology and the cellular microenvironment.

Although *hiPSCGEM01* primarily supports the optimization of in vitro experiments, its predictive power is constrained by incomplete knowledge of hiPSC metabolic networks. Limitations include the partial representation of complex in vivo systems, encompassing tissue-specific signaling, heterogeneity, and immune interactions. Missing or inaccurate pathway data may bias predictions and reduce clinical relevance. Ethical aspects further underscore the need for caution, particularly in avoiding overinterpretation of computational results, ensuring patient safety, and addressing disparities in access to advanced technologies. As a preliminary framework, *hiPSCGEM01* also requires expansion through integration of larger and heterogeneous transcriptomic datasets, such as those provided by Carcamo-Orivé et al. [92], which account for sex, age, BMI, and different reprogramming methods.

While computational insights are valuable, experimental validation remains essential. Advanced techniques, including CRISPR-Cas9-based gene editing, will be critical to confirm predicted essential genes and metabolites, thereby refining the model’s accuracy. Coupling experimental data with computational outputs will aid in improving reprogramming efficiency, optimizing culture media, and deepening our understanding of hiPSC physiology. Furthermore, traditional GEMs are static; thus, integrating them with dynamic modeling approaches to capture time-dependent changes in metabolite levels and fluxes represents a crucial next step [93,94].

In summary, *hiPSCGEM01* represents an important step toward unraveling the metabolic landscape of hiPSCs. By combining computational modeling with experimental validation, future efforts should focus on refining and expanding the model to optimize culture conditions, identify novel therapeutic targets, and engineer hiPSCs with tailored metabolic phenotypes, thereby advancing regenerative medicine and hiPSC-based therapies.

## Figures and Tables

**Table 1:** Genome-expression dataset selected from GEO data bank, and employed to reconstruct the hiPSC model.

GEO ID	Authors	Description	Series
GSE14711	Soldner, F. et al. [46]	hiPSCs were generated by reprogramming fibroblasts derived from 5 Parkinson’s disease patients.	GSM367219 GSM367240 GSM367241 GSM367242 GSM367243 GSM367244 GSM367245 GSM367258
GSE9865	Lowry, W. E. et al. [47]	hiPSC were generated through the reprogramming of dermal fibroblasts using various methodologies.	GSM249028 GSM249095 GSM249096 GSM249137

**Table 2:** Top 32 results from the Metabolites Search Enrichment Analysis (MSEA), performed on the essential metabolites by using the MetaboAnalyst tool. These metabolic pathways and reactions play crucial roles in maintaining hiPSC pluripotency, supporting cell growth, and providing the necessary building blocks for differentiation into specific cell types.

Pathway	Essential Metabolites List
Fatty Acid Elongation In Mitochondria	NADP, Palmitic acid, NADPH, NAD, Octanoyl-CoA, Acetyl-CoA, Coenzyme A, NADH, Water, (2E)-Dodecenoyl-CoA, (2E)-Decenoyl-CoA, Decanoyl-CoA (n-C10:0CoA), Hydrogen Ion
Fatty Acid Metabolism	Adenosine monophosphate, L-Carnitine, Palmitic acid, Pyrophosphate, Adenosine triphosphate, NAD, Octanoyl-CoA, Acetyl-CoA, Coenzyme A, NADH, Carbon dioxide, Water, (2E)-Decenoyl-CoA, Decanoyl-CoA (n-C10:0CoA), Hydrogen Ion
Alpha Linolenic Acid and Linoleic Acid Metabolism	Linoleic acid, Arachidonic acid, Alpha-Linolenic acid, Eicosapentaenoic acid, Cis-(8,11,14,17)-Eicosatetraenoic acid, Docosahexaenoic acid, Adrenic acid, (8,11,14)-Eicosatrienoic acid, Gamma-Linolenic acid, Docosapentaenoic acid, Stearidonic acid
Transfer of Acetyl Groups into Mitochondria	NADP, NADPH, Adenosine triphosphate, NAD, Acetyl-CoA, ADP, Coenzyme A, NADH, Carbon dioxide, Water, Hydrogen Ion
Glycerolipid Metabolism	Glycerol 3-phosphate, NADP, Palmitic acid, NADPH, Adenosine triphosphate, NAD, ADP, Coenzyme A, NADH, Water
Plasmalogen Synthesis	Cytidine monophosphate, NADP, NADPH, Stearic acid, NAD, Stearoyl-CoA, Oxygen, Citicoline, Coenzyme A, NADH, Water, Hydrogen peroxide
Glucose-Alanine Cycle	L-Glutamic acid, L-Alanine, NADP, NADPH, NAD, NADH, Water, Hydrogen Ion
Mitochondrial Beta-Oxidation of Short Chain Saturated Fatty Acids	Adenosine monophosphate, L-Carnitine, Pyrophosphate, Adenosine triphosphate, NAD, Octanoyl-CoA, Acetyl-CoA, Coenzyme A, NADH, Water, Hydrogen Ion
Urea Cycle	Adenosine monophosphate, L-Glutamic acid, L-Alanine, L-Aspartic acid, Pyrophosphate, L-Arginine, Adenosine triphosphate, L-Glutamine, NAD, ADP, NADH, Carbon dioxide, Water
Pyruvate Metabolism	Adenosine monophosphate, NADP, NADPH, Pyrophosphate, Adenosine triphosphate, NAD, Malonyl-CoA, Acetyl-CoA, Guanosine triphosphate, ADP, Coenzyme A, NADH, Carbon dioxide, Water, Hydrogen Ion
Glutamate Metabolism	Adenosine monophosphate, Glycine, L-Glutamic acid, L-Alanine, L-Aspartic acid, NADP, NADPH, Pyrophosphate, Adenosine triphosphate, L-Cysteine, L-Glutamine, NAD, Acetyl-CoA, ADP, Coenzyme A, NADH, Carbon dioxide, Water, Hydrogen Ion
Ammonia Recycling	Adenosine monophosphate, Glycine, L-Glutamic acid, L-Asparagine, L-Histidine, L-Serine, L-Aspartic acid, Pyrophosphate, Adenosine triphosphate, L-Glutamine, NAD, ADP, NADH, Carbon dioxide, Water
Glycine and Serine Metabolism	Adenosine monophosphate, Glycine, L-Glutamic acid, L-Alanine, L-Threonine, L-Serine, Pyrophosphate, L-Arginine, Adenosine triphosphate, L-Cysteine, L-Methionine, NAD, Acetyl-CoA, ADP, Oxygen, Coenzyme A, NADH, Carbon dioxide, Water, Hydrogen peroxide
Pyrimidine Metabolism	Deoxycytidine, Cytidine triphosphate, Cytidine monophosphate, NADP, NADPH, Pyrophosphate, Uridine triphosphate, Uridine $5^{\prime }$-monophosphate, Uridine $5^{\prime }$-diphosphate, Adenosine triphosphate, L-Glutamine, dCTP, dCMP, dCDP, dTDP, ADP, Thymidine $5^{\prime }$-triphosphate, Carbon dioxide, Water
Glutathione Metabolism	Glycine, L-Glutamic acid, L-Alanine, NADP, NADPH, Adenosine triphosphate, L-Cysteine, ADP, Water, Hydrogen peroxide
Pantothenate and CoA Biosynthesis	Adenosine monophosphate, Cytidine triphosphate, Cytidine monophosphate, Pyrophosphate, Adenosine triphosphate, L-Cysteine, ADP, Coenzyme A, Carbon dioxide, Water
Phytanic Acid Peroxisomal Oxidation	NADP, NADPH, Pyrophosphate, Adenosine triphosphate, NAD, Acetyl-CoA, ADP, Oxygen, Coenzyme A, NADH, Carbon dioxide, Water
Mitochondrial Beta-Oxidation of Medium Chain Saturated Fatty Acids	Adenosine monophosphate, Pyrophosphate, Adenosine triphosphate, Dodecanoic acid, NAD, Octanoyl-CoA, Acetyl-CoA, Coenzyme A, NADH, Water, (2E)-Dodecenoyl-CoA, (2E)-Decenoyl-CoA, Decanoyl-CoA (n-C10:0CoA), Hydrogen Ion
Cardiolipin Biosynthesis	Cytidine triphosphate, Cytidine monophosphate, Glycerol 3-phosphate, Pyrophosphate, NAD, Coenzyme A, NADH, Water, Hydrogen Ion
Mitochondrial Beta-Oxidation of Long Chain Saturated Fatty Acids	Adenosine monophosphate, L-Carnitine, Pyrophosphate, Adenosine triphosphate, Stearic acid, NAD, Stearoyl-CoA, Acetyl-CoA, Coenzyme A, NADH, Water, Hydrogen Ion
Ethanol Degradation	Adenosine monophosphate, NADP, NADPH, Pyrophosphate, Adenosine triphosphate, NAD, Acetyl-CoA, Oxygen, Coenzyme A, NADH, Water, Hydrogen peroxide, Hydrogen Ion
Arginine and Proline Metabolism	Adenosine monophosphate, Glycine, L-Glutamic acid, L-Proline, L-Aspartic acid, NADP, NADPH, Pyrophosphate, L-Arginine, Adenosine triphosphate, NAD, ADP, Oxygen, NADH, Carbon dioxide, Water, Hydrogen peroxide, Hydrogen Ion
Nicotinate and Nicotinamide Metabolism	Adenosine monophosphate, L-Glutamic acid, NADP, NADPH, Pyrophosphate, Adenosine triphosphate, L-Glutamine, NAD, ADP, Oxygen, NADH, Carbon dioxide, Water, Hydrogen peroxide, Hydrogen Ion
Beta-Alanine Metabolism	L-Glutamic acid, L-Histidine, L-Aspartic acid, NADP, NADPH, NAD, Acetyl-CoA, Oxygen, Coenzyme A, NADH, Carbon dioxide, Water, Hydrogen peroxide, Hydrogen Ion
Purine Metabolism	Adenosine monophosphate, Glycine, L-Glutamic acid, L-Aspartic acid, NADP, NADPH, Pyrophosphate, Adenosine triphosphate, L-Glutamine, NAD, Guanosine triphosphate, ADP, Oxygen, dGTP, NADH, dADP, Deoxyadenosine triphosphate, Carbon dioxide, Water, Hydrogen peroxide
Histidine Metabolism	Adenosine monophosphate, L-Glutamic acid, L-Histidine, NADP, NADPH, Pyrophosphate, Adenosine triphosphate, NAD, ADP, Oxygen, NADH, Carbon dioxide, Water, Hydrogen peroxide, Hydrogen Ion
Phosphatidylethanolamine Biosynthesis	Cytidine triphosphate, Cytidine monophosphate, L-Serine, Pyrophosphate, Adenosine triphosphate, ADP, Carbon dioxide, Hydrogen Ion
Aspartate Metabolism	Adenosine monophosphate, L-Glutamic acid, L-Asparagine, L-Aspartic acid, Pyrophosphate, L-Arginine, Adenosine triphosphate, L-Glutamine, Guanosine triphosphate, Oxygen, Carbon dioxide, Water, Hydrogen peroxide
Lysine Degradation	L-Glutamic acid, L-Lysine, NADP, NADPH, NAD, Acetyl-CoA, Oxygen, Coenzyme A, NADH, Carbon dioxide, Water, Hydrogen peroxide
Propanoate Metabolism	Adenosine monophosphate, L-Glutamic acid, Pyrophosphate, Adenosine triphosphate, L-Valine, NAD, Malonyl-CoA, Acetyl-CoA, ADP, Coenzyme A, NADH, Carbon dioxide, Water, Hydrogen Ion
Phosphatidylcholine Biosynthesis	Cytidine triphosphate, Cytidine monophosphate, Pyrophosphate, Adenosine triphosphate, ADP, Phosphorylcholine, Carbon dioxide, Hydrogen Ion
Nucleotide Sugars Metabolism	Pyrophosphate, Uridine triphosphate, Adenosine triphosphate, NAD, ADP, Glucose 6-phosphate, NADH, Carbon dioxide, Water
Methionine Metabolism	Adenosine monophosphate, Glycine, L-Serine, Pyrophosphate, Adenosine triphosphate, L-Cysteine, L-Methionine, NAD, Oxygen, NADH, Carbon dioxide, Water, Hydrogen peroxide
Cysteine Metabolism	Adenosine monophosphate, L-Glutamic acid, Pyrophosphate, Adenosine triphosphate, L-Cysteine, NAD, ADP, Oxygen, NADH, Water

## Data Availability

The SBML file for the *hiPSCGEM01*, validated using the online SBML Validator, has been deposited in the Biomodels repository of the European Bioinformatics Institute under the unique identifier MODEL2407190010. Additionally, the model is accessible through a dedicated git-hub repository.
